# Semisupervised Deep Learning for the Detection of Foreign Materials on Poultry Meat with Near-Infrared Hyperspectral Imaging

**DOI:** 10.3390/s23167014

**Published:** 2023-08-08

**Authors:** Rodrigo Louzada Campos, Seung-Chul Yoon, Soo Chung, Suchendra M. Bhandarkar

**Affiliations:** 1 School of Computing, University of Georgia, Athens, GA 30602, USA; rlc@uga.edu (R.L.C.); suchi@uga.edu (S.M.B.); 2 U.S. National Poultry Research Center, Agricultural Research Service, U.S. Department of Agriculture, Athens, GA 30605, USA; 3 Department of Biosystems Engineering, Integrated Major in Global Smart Farm, Research Institute of Agriculture and Life Sciences, Seoul National University, Seoul 08826, Republic of Korea; soochung@snu.ac.kr

**Keywords:** chicken breast fillets, deep learning, foreign material detection, generative adversarial network, hyperspectral imaging, near infrared, semisupervised learning

## Abstract

A novel semisupervised hyperspectral imaging technique was developed to detect foreign materials (FMs) on raw poultry meat. Combining hyperspectral imaging and deep learning has shown promise in identifying food safety and quality attributes. However, the challenge lies in acquiring a large amount of accurately annotated/labeled data for model training. This paper proposes a novel semisupervised hyperspectral deep learning model based on a generative adversarial network, utilizing an improved 1D U-Net as its discriminator, to detect FMs on raw chicken breast fillets. The model was trained by using approximately 879,000 spectral responses from hyperspectral images of clean chicken breast fillets in the near-infrared wavelength range of 1000–1700 nm. Testing involved 30 different types of FMs commonly found in processing plants, prepared in two nominal sizes: 2 × 2 mm^2^ and 5 × 5 mm^2^. The FM-detection technique achieved impressive results at both the spectral pixel level and the foreign material object level. At the spectral pixel level, the model achieved a precision of 100%, a recall of over 93%, an F1 score of 96.8%, and a balanced accuracy of 96.9%. When combining the rich 1D spectral data with 2D spatial information, the FM-detection accuracy at the object level reached 96.5%. In summary, the impressive results obtained through this study demonstrate its effectiveness at accurately identifying and localizing FMs. Furthermore, the technique’s potential for generalization and application to other agriculture and food-related domains highlights its broader significance.

## 1. Introduction

Foreign material (FM) in the poultry industry is one of the important food safety concerns. FMs, such as small pieces of plastic, rubber, and fabric, can be accidentally and randomly deposited on the product’s surface or embedded in the product during poultry processing. The final products contaminated with these FMs may pose a severe risk to the health and safety of consumers and place a financial burden on the poultry processors affected by product recalls. The sources of the FMs found in poultry products are primarily the processing equipment, personal protective equipment (PPE), and various materials used during poultry processing. Due to the massive volume of meat production in poultry processing plants, a manual inspection of all products for the presence of surface FMs is unfeasible. Therefore, an automated solution to detect the FMs found on the surface of meat is highly desirable due to the limitation of the existing sensing technologies.

Previous research proposed many nondestructive sensing techniques to detect FMs in food, such as metal detection, X-rays, thermal imaging, spectroscopy, magnetic resonance imaging, and computer vision [1]. Metal detectors and X-ray machines are widely adopted in the poultry industry for FM detection. X-ray technology has effectively detected broken bones embedded in poultry meat [2,3]. However, X-ray imaging has shortcomings in detecting low-density materials such as plastic, rubber, and fabric [4], which are commonly used in poultry processing equipment and PPE. Metal detectors have the same issue as X-ray machines when detecting low-density materials. Thermal imaging can be affected by the temperature of the surrounding environment [5]. Terahertz spectroscopy’s nonionizing radiation and low photon energies suffer from their limited ability to penetrate water [6,7], which is highly prevalent in poultry meat (~75% water in the muscles) [8]. Color-based computer vision techniques can also suffer from similarities in color between FM and meat samples [9].

Hyperspectral imaging (HSI) technology has also been applied for FM detection in agricultural and food applications [10,11,12]. A support vector machine (SVM) model for hyperspectral image classification was proposed to detect microplastics in the intestinal tracts of fish [13]. An SVM and a backpropagation neural network were studied to detect FMs from multispectral images of pickled and dried mustard [14]. A region proposal network (RPN) and a 3D convolutional neural network (CNN) were combined to detect FMs from hyperspectral images of red meat [15]. The study most closely related to this paper is the sensor fusion work of visible near-infrared (NIR) and short-wave infrared (SWIR) HSI modalities to detect FMs on poultry meat through chemometrics and shallow machine learning [11].

Deep learning (DL) models such as CNNs have proven powerful for hyperspectral image classification and analysis with their ability to automatically learn hierarchical, complex, and nonlinear representations of data [16]. This ability is achieved through deep neural networks (DNNs) consisting of multiple layers of interconnected nodes that are trained to extract increasingly abstract and informative features from the data so that the DNNs can capture the underlying patterns and relationships in the data and can make accurate and robust predictions [17]. In contrast, traditional chemometrics and/or machine learning approaches often rely on hand-crafted features and models designed based on domain knowledge and assumptions, which can be limited and prone to error [18]. Another advantage of DL models is that they can handle large high-dimensional data such as hyperspectral images. Several studies have reported that the use of DL in HSI improved the classification accuracy, computational efficiency, and robustness to noise and solved other challenges [13,19,20]

Recently, a generative adversarial network (GAN) was used for anomaly detection [21,22] in an unsupervised way. Jiang et al. [21] proposed a GAN-based anomaly detection model for hyperspectral images in remote sensing, where anomaly suppressed hyperspectral images were reconstructed and a detection map was predicted by using differences between the original and reconstructed images. Another study also utilized a GAN for anomaly suppression by background reconstruction [23]. The aforementioned unsupervised GAN models for anomaly detection adopted a data distribution assumption that anomalies always reside in low-density areas and can be distinguished from the background not only in a reduced low-dimensional representation space but also in the reconstruction space. This data distribution assumption did not directly apply to our research problem because FMs could be observed only at random times and locations. In addition, our preliminary study suggested that the performance of these GAN-based unsupervised approaches was relatively poor when it came to detecting FMs on poultry meat because the prediction of the low-density areas was not always accurate. Unsupervised learning also increases the computational complexity due to the need for an extensive training set, which may decrease the model’s performance. Supervised FM detection could be an alternative to unsupervised FM-detection models. However, supervised learning models may require both normal chicken and abnormal FM data for the model training and often can be difficult to calibrate in practice due to the time and cost of collecting high-quality annotated FM data. A recent study demonstrated that a semisupervised multichannel GAN model could address the inadequacy of labeled data in medical image analyses [24]. Similarly, a semisupervised model using a GAN can be useful in practice for FM detection because only normal (clean) chicken data can be used for training, which is cost effective because of the abundance of normal data. Even though the 1D spectral data of chicken meat were easily obtainable, the training dataset would be imbalanced between spectrally different components of chicken meat, such as the muscle tissue and fatty connective tissue.

The architecture of the original GAN [25] and its most common variations [26,27] is composed of a generator to generate fake samples and a discriminator to determine whether the generated sample is a fake or real one. Schönfeld et al. [28] proposed a new GAN model with a 2D U-Net-based discriminator adopting an encoder–decoder network with bottleneck and skip connections connecting the encoder and decoder for the localized segmentation of fake and real pixels (or regions) and the global classification of fake and real image samples. This new GAN model increased the quality of the generated 2D color images while increasing the distinction between the real and generated images.

This study is based on several hypotheses: (1) it is feasible to develop a semisupervised FM-detection model by using hyperspectral imaging; (2) the performance of the FM-detection model is affected by the spectral heterogeneity between the muscle tissue and fatty tissue; (3) integrating a 1D U-Net discriminator into a GAN architecture enhances the model’s performance; and (4) there exists an optimal spectral range suitable for the FM-detection model.

The specific objectives of this study are (1) to develop a semisupervised GAN model for FM detection by incorporating a 1D U-Net discriminator into the GAN model, (2) to improve the FM-detection performance by training two independent GAN models for spectral signatures unique to the muscle tissue and fatty tissue in chicken meat, and (3) to conduct a comparative analysis of the model’s performance by using different wavelength ranges. Additionally, the main contributions of this paper are as follows: (1) a semisupervised training strategy eliminated the need for FM data collection during the training of the FM-detection models, (2) NIR HSI (1000–1700 nm) was combined with the DL-based multichannel GANs and a 1D U-Net GAN discriminator, and (3) the outputs of the 1D spectrum-based GAN model were aggregated and spatially mapped onto a 2D prediction image to allow for FM detection at object and image levels.

Following this Introduction, the Materials and Methods section provides a detailed description of the hyperspectral dataset of chicken fillets used and the proposed semisupervised deep learning model. This includes the explanation of the Gaussian mixture model-based spectral clustering, generative adversarial network architecture, postprocessing steps for foreign material detection, and model performance evaluation. The Results and Discussion section presents the performance of the foreign material detection models at the pixel, blob, and image levels. Finally, the conclusion summarizes the key findings and implications of this work.

## 2. Materials and Methods

### 2.1. Chicken Fillet and Foreign Material Samples

A total of 12 raw halved boneless skinless broiler breast fillets (pectoralis major muscle) and 30 different types of FMs were used for this study. The 12 fillets were divided into a training set of 6 and a test set of 6. Both surfaces (the skin and bone sides) of each fillet were imaged by using a short-wave infrared (SWIR) HSI system in the wavelength range from 1000 nm to 2500 nm with and without FMs. All 12 clean fillets were imaged first, and then each fillet surface was contaminated with the FM samples and imaged. The background of the images was a food-grade polyurethane conveyor belt (blue). There were two types of sizes for the FM pieces for imaging: 2 mm × 2 mm (small) and 5 mm × 5 mm (large), approximately. Details of the FM samples were described in the previous work [11]. The FMs used in this study belonged to one of three FM groups: polymer (rubber and plastic), metal (aluminum and stainless steel), and wood.

### 2.2. SWIR Hyperspectral Image Acquisition and Preprocessing

The SWIR hyperspectral images were acquired with a push broom HSI system equipped with in-house developed C++ application software, a mercury–cadmium–telluride (MCT) camera, a spectrograph, illuminators (two 50 W tungsten halogen lamps), and a motorized linear stage for line scanning in a diffuse reflectance mode. They were preprocessed as described in the previous work [11]. In this section, key highlights are provided with updated information next.

The C++ application software controlled the motorized linear stage for the line scans and the MCT camera to acquire and save hyperspectral images. The intensity calibration of an acquired hyperspectral image was conducted with a dark current and white references recorded as horizontal stripes on top of each image. A lens cap was put on while collecting the dark current references (60–70 lines) at the beginning, and a white reference standard with dimensions of 1″ × 12″ and a 99% diffuse reflectivity (Fluorilon FW-99, Avian Technologies, New London, NH, USA) was used to collect additional 60–70 white lines right after the current dark lines. The horizontal stripes of the dark current (*DC*) and white (*WH*) references were averaged when calculating the intensity calibration to obtain a percent reflectance value (*R_c_*) from a measured digital number (*DN*) at each pixel via $R_{c}=\frac{DN-{DC}_{average}}{{WH}_{average}-{DC}_{average}}\times 100\left(\%\right).$

Each calibrated hyperspectral image was denoised with a Savitzky–Golay (SG) smoothing filter (the 4th order polynomial with a kernel size of 7) followed by the removal of the top reference slices spatially and the cropping of the wavelength range to 1000–2500 nm (199 bands) spectrally.

**Figure 1:**
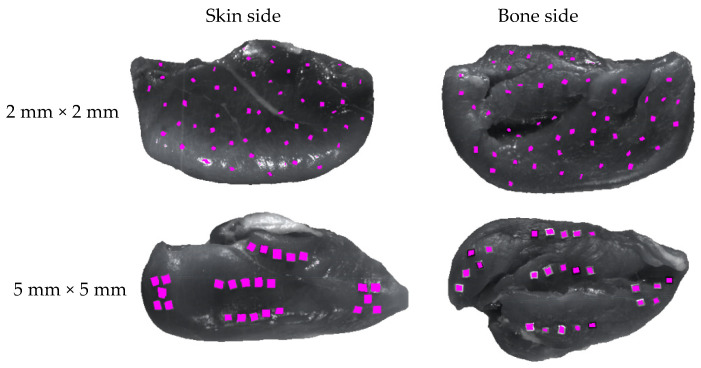
Waveband images of the skin (**left**) and bone (**right**) side of a fillet at 1000 nm with contaminated foreign materials (magenta color) in two different sizes.

The foreground region corresponding to the fillet was automatically detected and segmented with a thresholding algorithm as described in the previous work [11]. The FMs on the images were labeled with the region-of-interest (ROI) tool in ENVI (v4.8, L3Harris Geospatial, Broomfield, CO, USA). When evaluating the model performance, these annotated pixels and ROIs were used as the ground truth. The binary labeling rule for FM detection assigned 0 to the meat and 1 to the FMs within the foreground area. On the other hand, the multilabeling rule for analyzing the model’s performance on material types assigned 1 to muscle, 2 to fat, 3 to any other normal features, and 4 to 33 to each type of the 30 FMs. Figure 1 shows two example waveband images at 1000 nm with large and small FMs that were overlaid with the ground-truth ROIs (magenta color). The SWIR 1000–2500 nm wavelength range with 199 wavebands was reduced to the NIR 1000–1700 nm (exactly 997.2–1698.9 nm) range with 95 wavebands. In this study, both the SWIR and NIR wavelength ranges as well as the data dimensionality reduction by principal component analysis (PCA) were compared with each other in terms of their FM-detection performance, and the best one was selected.

### 2.3. Dataset

A total of 48 SWIR hyperspectral images were collected for this study. Each hyperspectral image had three dimensions along the X (horizontal), Y (vertical), and λ (wavelength) axes, and each image was flattened to a 2D array, where a 1D spectral row vector (1xλ) at each pixel position (x,y) was put into a single row in the 2D array. Note that only the spectral vectors at the pixels on the foreground fillet region were added to the 2D array. A single-column vector of each pixel’s class membership, such as the meat, fat, and FM type, was also added to the 2D array as a class label vector. The spatial coordinates (x,y) of each 1D spectral vector were kept such that the outputs of the deep learning model using 1D spectral vectors would be remapped into the original image locations. Then, these 2D arrays from all the hyperspectral images were concatenated (stacked) vertically into a skinny-and-tall matrix of 2D arrays, constituting the dataset used for the prediction model development and evaluation. This dataset was divided for training and testing according to the following rule: (1) 12 images of two surfaces of six clean fillets were set aside for training, and (2) the other 12 clean images of the remaining six fillets and 24 images of all of the contaminated twelve fillets were used for testing.

In summary, the number of hyperspectral images used for training was 12 (25%) without FMs while the number of hyperspectral images used for testing was 36 (75%), including 12 clean images without FMs and 24 contaminated images with FMs. The dimensions of the spectral data matrix used for training were 865,583 (height: number of spectral responses) × the number of bands (width), where the number of bands was different based on the wavelength range used, i.e., 199 for SWIR, 95 for NIR, and 3 for the PCA-based model. Each SWIR hyperspectral image was utilized to generate a corresponding NIR hyperspectral image. Additionally, from the NIR hyperspectral image, three PCA score images, derived from the three principal components (PCs), were created. The dimensions of the spectral data matrix used for testing were 2,607,898 (height: the number of spectral responses) × the number of bands (width), where the number of bands was the same as that of the training set.

### 2.4. Semisupervised Deep Learning Model for Foreign Material Detection

**Figure 2:**
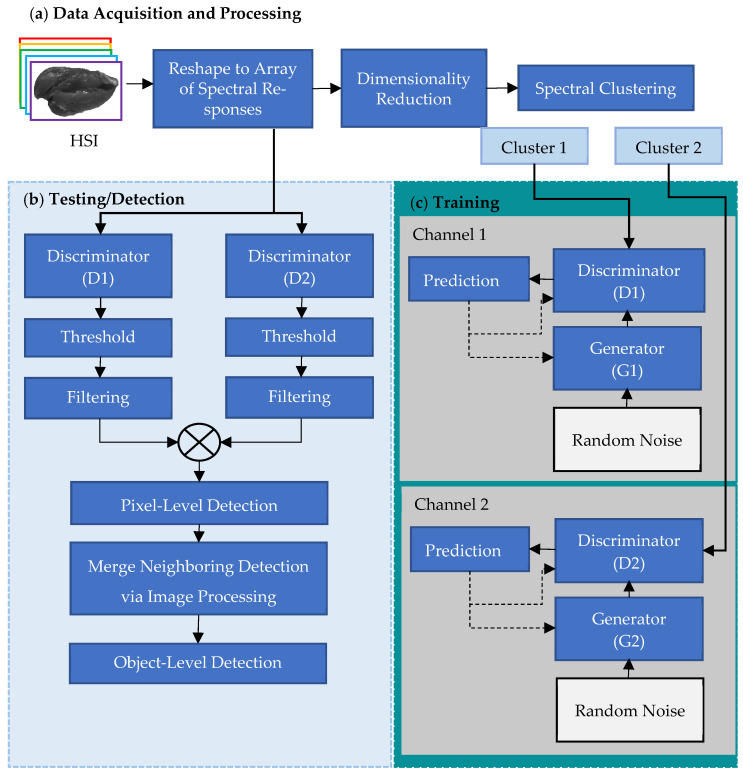
Schematic of the proposed FM-detection method, encompassing (**a**) hyperspectral data acquisition and preprocessing, (**b**) inferencing (testing) and FM detection, and (**c**) individual GAN-channel training.

The developed semisupervised DL model assumed that (1) the clean chicken meat was only necessary to make the model learn the distribution of spectral responses during training, and (2) when a spectral response of FM was presented to the trained model, a prediction error would be much higher than when a normal spectral response was presented. To realize these assumptions, a DL model was designed to detect the FMs at each pixel without its spatial context and to map the pixel-level detection results back to the image domain via postprocessing. Technically, the DL model was constructed with two-channel GAN modules featuring 1D U-Net discriminators. The inputs to these GAN modules were derived from the outputs of two 1D spectral clustering modules. The model’s input was a 1D spectral vector at position (x,y) while the output was a binary value (1: FM or 0: normal) assigned to each pixel position (x,y). The architecture and workflow of the DL model for the automatic detection of FMs are shown in Figure 2.

#### 2.4.1. Unsupervised Spectral Clustering

Diffuse reflectance spectra of chicken breast fillets are inhomogeneous [29] because poultry meat consists of various compositions with different quantities, including water (~75%), protein (~20%), fat (~3%), and other chemical nutrients [8,30]. These components in poultry meat respond spectrally differently in the NIR wavelengths [31,32,33]. This inhomogeneity in the spectral responses would make a machine learning model more complex compared to the case of homogeneous spectral responses because the features to learn from inhomogeneous spectral responses would be more complex and varied.

This study tackled this issue by automatically splitting image pixels with different spectral characteristics into spectrally more homogenous subgroups via unsupervised clustering by utilizing the Gaussian mixture model (GMM) [34,35]. The GMM is based on the characterization of a heterogenous input data distribution with a linear mixture of unimodal Gaussian distributions and has been used in many different HSI applications, such as hyperspectral image segmentation [36,37], the monitoring of saline vegetation [38], and anomaly detection [39]. Given the input data vectors in an *n*-dimensional space and the number of clusters *K*, the GMM estimated the distribution of a data vector **x** with *K* unimodal Gaussian distributions that were linearly mixed in the following equation:

(1) $$P\left(x\right)=\sum_{k=1}^{K}w_{k}N\left(x|\mu_{k},\Sigma_{k}\right)\;\;s.t.\;\;\sum_{k=1}^{K}w_{k}=1$$

where $N\left(x|\mu_{k},\Sigma_{k}\right)$ is the *k*th normal distribution with a mean of $\mu_{k}$ and a covariance matrix of $\Sigma_{k}$, and $w_{k}$ is the weight of the *k*th Gaussian distribution. The shape of the estimated normal distribution was modeled with two parameters, $\mu_{k}$ and $\Sigma_{k}$. Expectation maximization (EM) was used to estimate the parameters of $\mu_{k}$, $\Sigma_{k}$, and $w_{k}$ for each of the *k* normal distributions that would fit the data best.

In this study, *K* = 2 was chosen as the number of spectrally homogeneous clusters. This choice was based on the findings of previous work, which showed notable differences in the average spectral responses between muscle tissue and fatty materials, such as excessive fat deposits and whitish connective tissue, observed on the surface of chicken breast fillets [11,29]. It is worth noting that poultry meat primarily comprises muscle tissue with a high water and protein content, whereas fatty tissues, including excessive fat deposits, whitish connective tissue, white stripes, and tendons, have a high fat content and are frequently observed on the surface of chicken breast fillets. Hence, this study hypothesized that the spectral responses of the pixels in the muscle and fatty tissues would be homogeneous within each spectral cluster but heterogeneous between the two spectral clusters. Accordingly, two distinctive GAN models were trained, each trained independently focusing on one of the two different spectral clusters (Figure 2). The rationale behind this approach was to effectively capture the inherent differences between muscle and fatty tissues in chicken breast fillets through the training of separate GAN models, ultimately leading to an improved spectral analysis and classification of the fillet surface.

#### 2.4.2. Generative Adversarial Network (GAN)

A GAN works as a two-player game with a generator and a discriminator that compete with each other [25]. The generator is modeled as a convolutional decoder network with upsampling-transposed convolutions (i.e., inverse convolutions) to generate fake samples while the discriminator is modeled as an encoder network with downsampling convolutions to classify/predict whether the generated sample is a fake or real one. The standard GAN is trained by minimizing the loss functions $L_{G}$ (a generator loss) and $L_{D}$ (a discriminator loss) in an alternating manner:

$$L_{G}=-E_{z}\left[log\left(D(G(z)\right)\right]$$

(2) $$L_{D}=-E_{x}\left[logD\left(x\right)\right]-E_{z}\left[log\left(1-D\left(G\left(z\right)\right)\right)\right]$$

where the generator *G* aims to map a latent variable *z* to a realistic sample, while the discriminator *D* aims to differentiate between the real sample *x* and the generated sample *G*(*z*).

Since the standard GAN [25] uses an encoder-based discriminator, many other GANs also use encoder-based generators [40,41,42]. Schönfeld et al. [28] proposed a 2D U-Net-based discriminator to increase the discrimination power between real and generated samples in 2D color images. The U-Net is a widely used deep neural network and has demonstrated a state-of-art performance in many complex image segmentation tasks [43,44,45] through a U-shaped architecture, where a contracting path in the encoder can extract global features while an expansive path in the decoder can provide local information combined with global contextual information from the encoder. More specifically, a 1D U-Net can process and classify spectral data through hierarchical feature extraction with convolutional layers. The initial convolutions detect basic patterns. Deeper layers extract more complex abstract features by combining prior outputs and learning intricate interband relationships and distinctive characteristics. The encoder compresses the data into highly representative features. The decoder then upsamples and concatenates these features for precise localization and classification. In this paper, a 1D U-Net discriminator is proposed instead of the 2D U-Net discriminator, and the proposed 1D U-Net-based discriminator (UnetD) is compared with the standard encoder-based discriminator, called the encoder-only discriminator (EncD), to determine their FM-detection performance.

**Figure 3:**
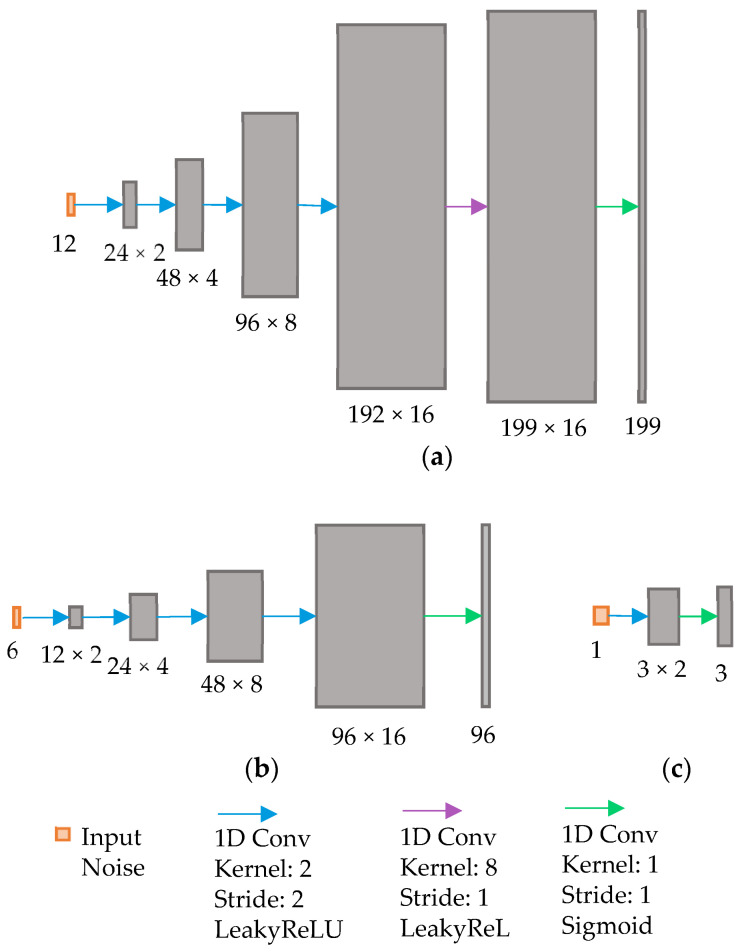
Architecture of the proposed GAN generator for different spectral output sizes for (**a**) SWIR (*n* = 199), (**b**) NIR (*n* = 96), and (**c**) PCA (*n* = 3) spectral data.

**Figure 4:**
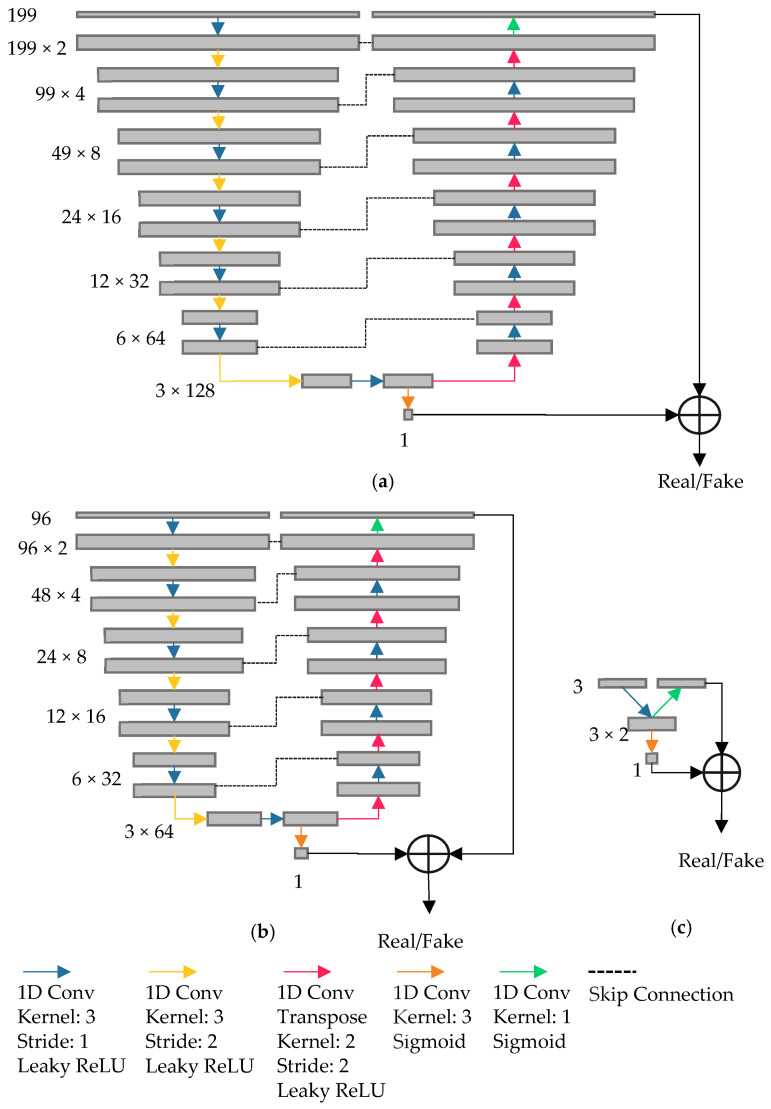
Architecture of the proposed GAN discriminator based on 1D U-Net for different spectral input sizes for (**a**) SWIR (*n* = 199), (**b**) NIR (*n* = 96), and (**c**) PCA (*n* = 3) spectral data.

Figure 3 and Figure 4 show the architectures of the GAN generators and discriminators used in this study. The input layer size was adjusted to accommodate the different dimensionalities of the SWIR (*n* = 199) and NIR (*n* = 96) and PCA (*n* = 3) spectral data. Note that the NIR spectral input vector was padded with one zero to make the length of the input layer 96 (Figure 3b and Figure 4b). Each generator consisted of an input layer, multiple 1D convolution layers with ReLu activation, and the last layer of the 1D convolution with sigmoid activation.

Each discriminator consisted of a contracting path and an expansive path, similar to U-Net. One layer in the contracting path of the discriminator-operated 1D convolution with kernel size 3 and stride 1 was followed by the 1D convolution with kernel size 3 and stride 2 for downsampling. The expansive path of the discriminator included 1D convolutional layers with kernel size 3 and stride 1 followed by 1D transposed convolutional layers of kernel size 2 and stride 2 for upsampling. The contracting and expansive paths were connected through skip connections (also known as residual connections) at each layer to stabilize the gradient updates by providing an uninterrupted gradient flow from the first to the last layer (Figure 4). There were two layers with a logistic sigmoid function as an activation function, one at the end of the contractive path and another one at the end of the expansive path. The classification losses from these two sigmoid activation layers were combined to generate a single loss map for FM detection. Note that the designed DL model provided a general global prediction over the input at the end of the contracting path, whereas the prediction from the model was more localized over wavelengths at the end of the expanding path. Similar to the generator, three versions of the discriminator were developed as well. Different versions were created to handle the SWIR, NIR, or 3PC input ranges with varying network depths (Figure 4).

#### 2.4.3. Training

The generator and discriminator of the GAN were trained separately but alternately in an iterative process one at a time. The generator was initialized to receive random noise as the input and to generate synthetic spectral data. It was trained to make this fake data increasingly realistic to fool the discriminator. Simultaneously, the discriminator was presented with a mix of real spectra from the ground truth data and artificial samples from the generator and were optimized to distinguish between the two accurately. The classification error of the discriminator was used to update the parameters of both networks through backpropagation, and reducing the error signaled improvements in the generator’s ability to mimic real data. Through this joint optimization of the adversary networks, the generator was trained to produce high-quality simulated data matching the distribution of real spectra.

#### 2.4.4. Postprocessing for FM Detection

**Figure 5:**
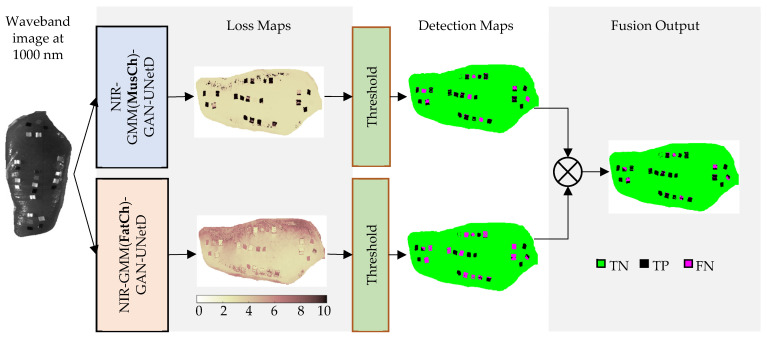
Example of inferencing to generate loss maps using GAN models at two clustering channels, thresholding the loss map in each channel, predicting detection images, and fusing two detection maps into a single detection map, starting from a single hyperspectral image.

During the inferencing using the test set, the discriminator of the GAN model was trained to predict the classification loss (error) of the input data, whose values were floating-point numbers. It is essential to mention that the trained generator was not utilized during inferencing as it was no longer required for this stage. The class labels (normal or FM) at the pixel, object, and image levels were determined through additional processing steps described next. Firstly, the predicted losses in a vector were reshaped into a loss map (called a prediction error image). The two-channel GAN models predicted their loss maps independently. Secondly, each loss map was binarized with global thresholding, producing a detection map with two values (0: normal and 1: FM). The global threshold value was selected by a histogram analysis based on the values in the loss maps. Then, a cumulative distribution function (CDF) was estimated from the obtained histogram. The maximum loss value for the normal meat samples in the training set was obtained when the CDF value became 1. The global threshold value was selected as the maximum loss value × 1.1 (a 10% increase from the maximum loss value for normal samples), such that the false positive rate (falsely classified as FM) was minimized at the expense of potentially missing FMs (increased false negatives), whose loss values were similar to the maximum loss value of the normal samples. Thirdly, 3 × 3 median filtering was applied to two detection maps to remove noise. Two denoised detection maps were then fused with a logical operator. After the logical OR and AND operators were compared, the AND operator was selected. Figure 5 shows the schematic of two-channel detection map prediction and fusion.

The spatial location and size of the predicted FM pixels in the fused detection map were analyzed at the object level by aggregating the predicted FM pixels into individual regions (called blobs) by using the 8-neighborhood system. The performance of the FM object detection was evaluated against the ground-truth FM ROIs in the test set. The final decision about the presence and absence of FMs was made per image by checking the prediction errors at the image level. In practice, the proposed hyperspectral imaging technique requires the automatic imaging of both sides of a fillet. The mechanized automatic flipping is feasible with an appropriate product flipper commonly used during manufacturing, which is out of the scope of this study.

### 2.5. Performance Evaluation

#### 2.5.1. Evaluation of GAN-Generated Spectral Data Quality

The quality of the GAN-generated data was evaluated with the Fréchet inception distance (FID) [46] and a mean spectral data analysis. The FID score is a widely adopted quantitative metric for assessing the performance of GAN models. It measures the similarity between the generated samples and real data, with a lower FID score indicating a higher quality and diversity of the simulated outputs. A low FID score implies that the generator has been effectively optimized to produce synthetic hyperspectral signatures that closely resemble the characteristics of the real fillet’s spectral data. The FID score was obtained to measure the distance between the generated and true spectral responses in the training set by using the following formula:

(3) $$\mathrm{FID}={\left\|\mu_{f}-\mu_{g}\right\|}_{2}^{2}+\mathrm{Tr}\left(\Sigma_{f}+\Sigma_{g}-2{\left(\Sigma_{f}\Sigma_{g}\right)}^{\frac{1}{2}}\right)$$

where ${\left||\cdot |\right|}_{2}^{2}$ denotes the Euclidean norm, $\mathrm{Tr}\left(\cdot \right)$ denotes the trace of a matrix, and µ and Σ are the mean and covariance of the generated (*f*) and ground-truth (*g*) feature vectors, respectively. The feature vectors were obtained from the pretrained inception network. A lower FID score indicates the better performance of the generative model. In the best-case scenario, when the FID score is 0, the synthetic data are an exact match to the real data.

#### 2.5.2. Pixel-Level FM-Detection Evaluation

The performance metrics used for the spectral pixel-level evaluation of the FM-detection models were the precision, recall, F1 score, and balanced accuracy (BACC) [47]. For the evaluation, the abnormal (FM or contaminated) pixel prediction was considered a positive outcome, whereas the normal (clean or uncontaminated) pixel was considered a negative outcome. All the predicted pixels in the test set were tallied for the number of true negatives (*TN*)—the number of correctly predicted normal pixels; true positives (*TP*)—the number of correctly predicted FM pixels; false negatives (*FN*)—the number of missed FM pixels (misclassified as normal); and false positives (*FP*)—the number of incorrectly predicted normal pixels (misclassified as FM). Given the *TP*, *FP*, and *FN*, the F1 score was calculated along with the precision and recall, as described below:

(4) $$Precision=\frac{TP}{TP+FP}=\frac{TP}{\#\;of\;all\;positives}$$

(5) $$Recall=\frac{TP}{TP+FN}=\frac{TP}{\#\;of\;all\;true\;FM\;pixels}=TPR$$

(6) $$F1\;score=2\cdot \frac{precision\times recall}{precision+recall}$$

The precision is the ratio of the number of correct FM predictions to the number of all pixels predicted as FM (correct or not). The recall, also called the sensitivity or true positive rate (*TPR*), is the ratio of the number of correct FM predictions to the number of all true FM pixels. The F1 score ranged from 0 (worst) to 1 (best)(equivalently, 0–100%). Note that the F1 score was calculated without the *TN*. Because the *TN* >> *TP*, there was a class imbalance problem in the dataset. The BACC accounted for this imbalance in two classes (# of meat pixels >> # of FM pixels) and was calculated according to the equation below:

(7) $$BACC=\frac{Sensitivity\left(=TPR\right)+Specificity\left(=TNR\right)}{2}=\frac{TPR+TNR}{2}$$

where specificity was defined as the true negative rate $\left(TNR=\frac{TN}{TN+FP}\right)$. The BACC values ranged from 0 (worst) to 1 (best) (equivalently, 0–100%).

#### 2.5.3. Object-Level FM-Detection Evaluation

The object-level evaluation started with image segmentation, where pixels predicted as FMs were spatially connected on 8-neighbors and the connected pixels were merged into individual segments called blobs or regions. Then, the intersection over union (IoU) [48] at each predicted FM object with an arbitrary shape was calculated as follows:

(8) $$IoU=\frac{Area\;of\;Overlap}{Area\;of\;Union}$$

where the overlap and union areas were computed between the prediction blob and ground-truth ROI. If the predicted blob did not intersect with the ground-truth ROI, the IoU was 0, whereas if they were completely matched, the IoU was 1. Given the IoU values from all the prediction blobs in the test set, the average IoU (AIoU) was obtained by averaging all the IoU values per model or FM group, whichever was appropriate. The AIoU was used to evaluate the performance of the FM detection according to the models and FM groups.

Given the IoU values computed for all the detected regions, a threshold *T* (e.g., 0.5) was applied such that a blob with an IoU ≥ *T* was considered a positive prediction and a blob with an IoU < *T* was considered a negative prediction. Then, the Jaccard Index (JI) and Overall Detection Accuracy (ODACC) were calculated with the following:

(9) $$Jaccard\;Index=\frac{{TP}_{b}}{{TP}_{b}+{FP}_{b}+{FN}_{b}}$$

(10) $$ODACC=\frac{Number\;of\;correctly\;detected\;blobs}{Number\;of\;ground-truth\;blobs}=\frac{{TP}_{b}}{{TP}_{b}+{FN}_{b}}$$

where *TP_b_* is the number of positive predictions, *FP_b_* is the number of negative predictions, and *FN_b_* is the number of missed ground-truth blobs. The IoU threshold value ranged from 0 to 1 with a step size of 0.1 [48]. The ODACC is the ratio between the number of ground-truth ROIs and the number of correctly predicted FM blobs, similar to the recall (or *TPR*) at the pixel level.

#### 2.5.4. Image-Level FM-Detection Evaluation

The performance at the image level was evaluated with the false positive rate, which measured how much the clean fillets were predicted incorrectly as the contaminated fillets. The detection performance at the image level was important because it measured how often clean fillets would be further processed due to a false positive error. For example, when only a single pixel is predicted as an FM from each clean fillet image, the pixel-level and object-level performance may show high F1 scores and IoU values, which are good. However, in this example, the false positive rate at the image level becomes 1, and all fillets will be declared to have FMs. Thus, in this study, the image-level false positive rate (IFPR) was calculated by counting how many images were predicted to have FMs (even a single pixel) incorrectly among all the clean fillet images.

#### 2.5.5. Summary of the Evaluated Models

The FM-detection models were evaluated and compared based on three main components (the input data type, spectral data clustering method, and GAN’s discriminator type) as described next. The input data types tested included (1) SWIR spectral data (1000–2500 nm); (2) NIR spectral data (1000–1700 nm); and (3) data obtained from the top-three PC scores in the PCA domain, denoted as SWIR, NIR, and 3PC. The 3PCs explained 98% of the variance in the SWIR data. The input spectral data clustering methods tested included (1) no clustering and (2) a Gaussian mixture model (GMM). When employing the GMM, two 1D spectral clusters were created, namely “FatCh” and “MusCh”, representing the fatty and muscle tissue channels, respectively. When specifying whether it pertained to the fatty tissue or muscle tissue, GMM(FatCh) or GMM(MusCh) was used to indicate the relevant clustering channel, respectively. The notation “GMM” alone denoted the proposed DL model using the GMM. Lastly, the discriminator types employed in the GAN were (1) UnetD representing a 1D U-Net discriminator and (2) EncD representing a 1D encoder-only discriminator. Various FM-detection models were evaluated by combining the aforementioned components with hyphens, such as NIR-GMM-GAN-UNetD.

The development and evaluation of these models were conducted on a Windows 11 machine with AMD Ryzen 9 5900HS and Nvidia RTX 3060 GPU (6 GB RAM). The software used was Python 3.10.10 and TensorFlow 2.12.0.

## 3. Results and Discussion

### 3.1. Generative Performance of GAN Models

The GAN models synthesized spectral data by using their generators during model training. The quality of the synthetically generated data was evaluated with mean spectral and FID score analyses.

**Figure 6:**
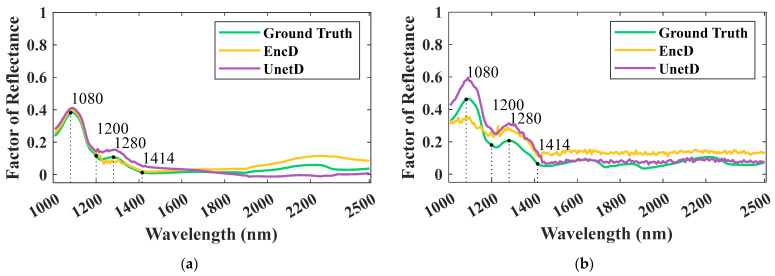
Synthetically generated spectra by GAN models for (**a**) muscle class channel and (**b**) fat class channel.

First, the comparison of the mean spectral data revealed the overall spectral differences between the muscle tissue and the fatty tissue, as depicted in Figure 6. This qualitative observation highlighted the spectral heterogeneity between these two tissue types, supporting the use of spectral clustering.

Second, Figure 6 shows the mean SWIR spectra generated by two GAN models with different discriminators (UnetD and EncD), each trained with clustered muscle (Figure 6a) and fat (Figure 6b) spectral groups. The synthetically generated data were compared with the ground-truth spectra. For the muscle spectral group, both the UnetD-based and EncD-based GAN models showed similar mean spectral responses to the ground truth from 1000 nm until approximately 1700 nm, with slight deviations in reflectivity compared to the ground truth mean spectrum between 1700 and 2500 nm. In the case of the fat spectral group, the UnetD-based GAN model outperformed the EncD-based model as it better captured the peaks and troughs at key wavelengths such as 1800, 1200, 1280, and 1414 nm. It is worth noting that the key NIR wavelengths at 1080 nm and 1414 nm are related to the water present in the meat samples, and 1200 nm and 1280 nm correspond to the C-H stretching modes from the lipid molecules within the meat samples. Compared to the muscle spectral clustering group, the quality of the generated spectra for the fat spectral sample clustering group was lower due to (1) the imbalance in the number of spectra between the muscle (high) and fat (low) groups and (2) the large spectral heterogeneity in the reflectivity of the fat class samples used for the clustering and GAN model training. It is also worth mentioning that, in the case of the fat data channel, the UnetD was more sensitive to variations across the bands compared to the EncD, which is why it forced the generator to better generalize the data, resulting in samples with less noise and more prominent peaks and troughs. The addition of the U-net decoding portion and local detection likely contributed to this improved smoothness shown in Figure 6b. In summary, the UnetD-based GAN model produced smoother and more accurate mean spectra compared to the EncD-based GAN model. The generated data at key wavebands also showed a closer similarity to the ground truth when NIR data were utilized.

Third, Table 1 shows the FID scores of the GAN-generated data compared to the ground-truth data in the SWIR, NIR, and PCA data spaces in the training set. The FID scores were calculated separately for the muscle- and fat-clustering-channel output groups, for the models trained without spectral clustering, and for the different GAN discriminators UnetD and EncD. Both the UnetD and EncD GAN discriminators trained with the muscle-channel SWIR spectral data achieved a higher similarity (lower FID scores: 2.31 for UnetD and 2.49 for EncD) when compared to the models trained without spectral clustering (2.57 for UnetD and 3.73 for EncD) and with the fat-channel output data (3.73 for UnetD and 3.10 for EncD). When reducing the data dimensionality from SWIR (1000–2500 nm) to NIR (1000–1700 nm), the similarity between the generated spectral responses and the ground truth improved from the average FID of 2.99 ((2.87 + 3.11)/2) for SWIR to 2.27 ((2.38 + 2.16)/2) for NIR. The PCA reduced the data distribution’s complexity and led to the best FID scores. It is important to note that while a low FID score could indicate a good generative performance, it did not necessarily imply the good detection performance of the 3PC GAN model.

The findings from the mean spectral analysis and FID scores suggest that the 1D U-Net discriminator would outperform the 1D encoder-only discriminator at FM detection and the NIR wavelength range would be more effective for the FM-detection model compared to the SWIR wavelength range. The results underscore the GAN model’s ability to effectively produce synthetic spectra that closely resemble real data, indicating its capability to enhance training datasets and facilitate improved performances in various applications. This study’s outcome aligns with previous research [49,50,51,52], highlighting the robustness and versatility of the GAN-UnetD approach at generating realistic spectral data for diverse hyperspectral analysis tasks.

### 3.2. Pixel-Level Detection Performance

**Figure 7:**
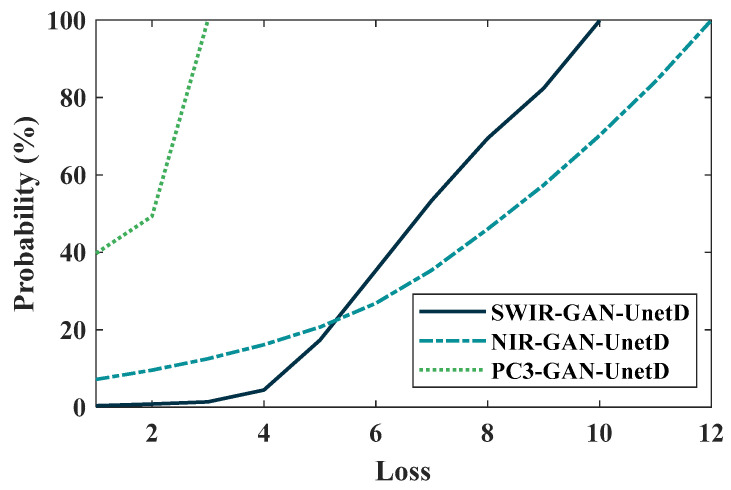
Cumulative distribution functions of training loss for three different GAN models (SWIR-GAN-UnetD, NIR-GAN-UnetD, and PC3-GAN-UnetD), utilized for threshold selection.

The precision and recall measured at the pixel level were dependent on the choice of the thresholding value during the prediction of a binary detection map from a loss map. In order to determine suitable threshold values for the NIR, SWIR, and 3PC models, the cumulative distribution function (CDF) of the training sample loss values was analyzed (as shown in Figure 7). The maximum training loss values corresponding to 100% CDF were determined to be 12 for NIR, 10 for SWIR, and 3 for 3PC. Based on these findings, the threshold values selected for the respective models were 13.2 for NIR, 11 for SWIR, and 3.3 for 3PC.

Table 2 presents the results of the pixel-level detection performance. The pixel-level performance of the FM-detection models was evaluated based on the type of discriminator used (UnetD and EncD), input data type (SWIR, NIR, and 3PC), and clustering approach (GMM and no clustering).

First, the comparison between the UnetD- and EncD-based GAN models showed that UnetD outperformed EncD, achieving better recall, F1-score, and BACC values, as predicted by the mean spectral data and FID score analyses. The GMM-GAN-UnetD models achieved the highest precision (99.7%), recall (85.2%), F1 score (91.3%), and BACC (92.6%). Notably, the NIR-based GMM-GAN-UnetD model showed an impressive 96.8% F1 score and 96.9% BACC. The UnetD models outperformed the EncD models significantly across all metrics, with UnetD achieving a 19.5% higher average recall, a 7.7% higher F1 score, and a 4.9% higher BACC compared to EncD. These findings underscore the effectiveness and superiority of the GMM-GAN-UnetD models, particularly when based on NIR spectral data, and demonstrate the substantial advantage of UnetD at achieving a superior FM-detection performance.

Second, the GMM-GAN-UnetD model trained with NIR data performed better than the models trained with SWIR and 3PC data, achieving the highest F1 score of 96.8% and BACC of 96.9%. The models trained with the SWIR data were the only ones presenting false positives (precision < 100%). When comparing the input types, the NIR-based UnetD models achieved a 7.3% higher recall and a 6.8% higher F1 score compared to the SWIR models. The 3PC models were the lowest, with an 80.8% F1 score for GMM-GAN-UnetD.

Furthermore, the GMM-GAN models showed a superior performance compared to the models trained without clustering. The GMM-GAN-UnetD models achieved an average F1 score of 91.3% and BACC of 92.6%, while the GAN-UnetD models without clustering achieved an average F1 score of 88.7% and BACC of 90.3%. The enhanced performance of the GMM compared to no clustering suggests that spectral clustering played a crucial role in reducing the interclass variability, specifically addressing the spectral heterogeneity hypothesis between the muscle and fatty tissues. By effectively grouping similar spectral signatures and separating different tissue types, the GMM aided with enhancing the GAN model’s ability to accurately distinguish and detect distinct components within the hyperspectral data. This reduction in interclass variability significantly contributed to the overall improvement in the FM-detection performance.

These results support this study’s hypotheses, including the hypotheses predicting that a semisupervised FM-detection model is feasible, the performance of the FM-detection model is affected by the spectral heterogeneity between the muscle tissue and fatty tissue, a 1D U-Net discriminator enhances the model’s performance, and the optimal wavelength range exists. The feasibility of semisupervised learning was successfully demonstrated by enhancing the previous supervised sensor fusion technique [7]. This was achieved by reducing two imaging modalities into a single imaging modality, substituting supervised learning with semisupervised learning, and exploring deep learning. The superior performance of the NIR-based model over the SWIR-based model implies that the data and system complexities can be reduced with an improved model performance. The 1D U-Net discriminator significantly improved the model’s performance over the traditional encoder-only discriminator. The results also provide strong evidence in favor of the proposed approach, highlighting the advantages of incorporating the 1D U-Net discriminator and leveraging the NIR wavelength range for enhanced FM detection in hyperspectral images.

### 3.3. Blob-Level Detection Performance

The rationale behind the blob-detection analysis is that even if every pixel of an FM is not accurately identified, detecting a significant portion of the FM can still enable flagging it as an FM. The performance of the blob detection is summarized in Table 3.

Most models exhibited similar scores for both the JI and ODACC due to the absence of false positives. A similar pattern observed at the pixel level was also observed at the blob level, where the models trained with GMM-GAN exhibited a superior performance compared to the models without clustering, and the UnetD models outperformed the EncD models. Upon analyzing the metrics aggregated over all the materials, UnetD achieved impressive JI scores ranging from 0.839 to 0.965, outperforming the EncD model with JI scores ranging from 0.551 to 0.922. These results indicate the superiority of UnetD at accurately segmenting and detecting different materials in the hyperspectral data, showcasing its effectiveness at achieving higher intersection-over-union values for the various materials considered in the evaluation.

However, it is important to highlight the distinctively poor performance of the 3PC models. When compared to the *TPR* of the NIR-GMM-GAN-UnetD model at the pixel level, the corresponding NIR model at the blob level demonstrated a performance improvement of over 2.6% in the ODACC (the *TPR* or recall at the blob level). Table 3 further demonstrates this discrepancy through the AIoU metric, indicating the poorer performance of the 3PC models. Despite blob detection being designed to enhance detection by classifying partially detected FM objects as detected objects, the 3PC models exhibited a significant number of false negatives in the wood and metal categories, resulting in the complete omission of many FM objects.

As mentioned above, the high generative performance of 3PC did not directly correlate to high FM detection. This low performance can be attributed to the possible intraclass variability between certain types of FM and clean training samples. Further, dimensionality-reduction algorithms, such as PCA, are typically linear techniques that cannot capture the complex nonlinear relationships present in high-dimensional data [53]. As a result, these techniques are often unable to preserve the essential features of the data, leading to a loss of information and performance degradation.

**Figure 8:**
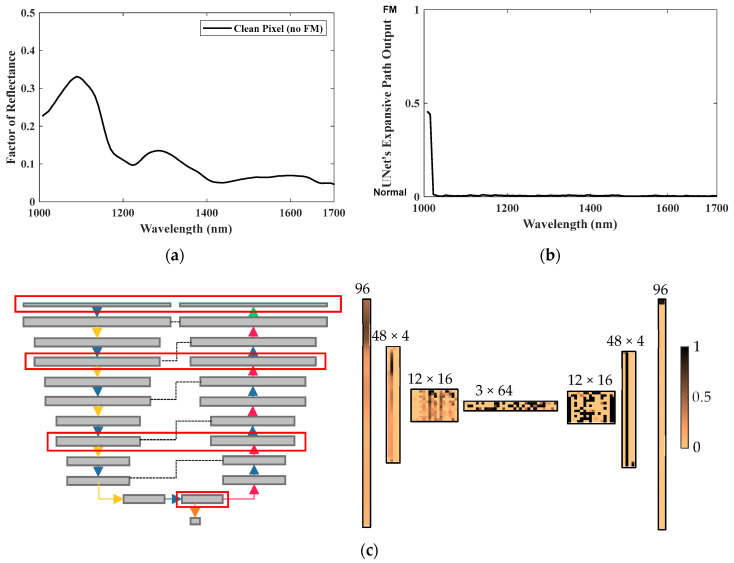
Localized detection power and layers of NIR-UnetD. (**a**) Input: a normal pixel’s spectrum; (**b**) output of UnetD’s expansive path in the decoder: localized prediction across the NIR wavelength range (0: clean, 1: FM); and (**c**) visual representation of intermediate layers. Note that the bandwise localized predictions were combined with the global prediction of the contracting path at the bottleneck in the last layer of the discriminator to predict the final loss value.

**Figure 9:**
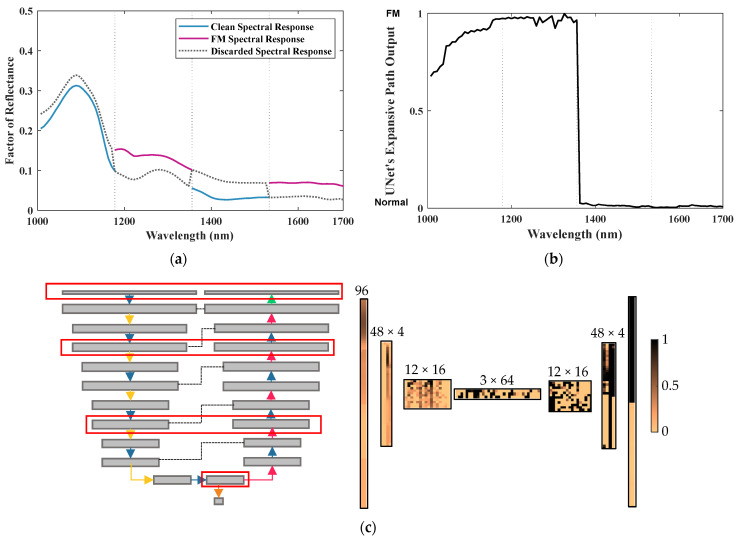
Localized detection power and layers of NIR-UnetD. (**a**) Input: a fabricated spectrum mixed with clean and FM spectrum responses; (**b**) output of UnetD’s expansive path: localized prediction across the NIR wavelength range (0: clean, 1: FM); and (**c**) visual representation of intermediate layers. Note that the bandwise localized predictions were combined with the global prediction of the contracting path at the bottleneck in the last layer of the discriminator to predict the final loss value.

Moreover, the proposed UnetD model offers an advantage over EncD by allowing for more precise decision making through its decoding path, enabling the classification of each spectral band individually. Figure 8 showcases the network’s classification of a spectral response of a clean meat sample, along with the intermediate layer features. Additionally, Figure 9 illustrates the network’s detection of a fabricated spectral response, which combines portions of a clean meat pixel’s spectral response with the spectral response of an FM. This fabricated example demonstrates the network’s performance under the challenging mixed-pixel effect. The spectral response was fabricated by alternating a clean spectral response with the spectral response of a semitransparent polymer FM.

The encoder portion of the discriminator classified the fabricated input as normal. However, the UnetD model provided a more detailed prediction after expanding the features through the decoding path. Notably, distinct detection patterns are observed among the four quadrants, with the most significant change occurring in the central portion. The first quadrant corresponded to the original clean spectral response, yet the prediction gradually shifted toward the FMs. This behavior can be attributed to the rolling convolution window, which utilizes neighboring bands for its calculations. In addition to outperforming a standard encoder EncD architecture, the proposed UnetD model exhibited robustness against the mixed-pixel effect, successfully detecting 100% of the polymer blobs present in the fabricated spectral response.

Moreover, Table 4 provides further evidence supporting the notion that GMM clustering helped reduce intraclass variability. The fusion of FatCh and MusCh detection yielded an increased performance. Specifically, SWIR and NIR FatCh demonstrated a relatively low detection performance of 0.514 AIoU for metallic materials, whereas NIR MusCh achieved a significantly higher detection performance of 0.881 AIoU for the same materials. However, when combining both detections, the final AIoU score improved by more than 5%. Similar effects were observed for wood, with an improvement of nearly 3%, while polymers exhibited the most significant improvement of 8.7%. The SWIR-GMM model demonstrated an average improvement of 6.86%, with wood achieving the highest improvement at 12.5%.

The results strongly support this study’s hypotheses, which align with the pixel-level results. The results demonstrate the feasibility of using the semisupervised GAN model for hyperspectral image-based FM detection. The findings also highlight the effectiveness of GMM clustering at enhancing the overall detection performance, particularly in scenarios where different classes of materials exhibit distinct characteristics. The substantial performance gains when fusing the FatCh and MusCh outputs indicate that the GMM clustering successfully reduced the intraclass variability, enabling more consistent material detection. If high variability remained within each class, combining the independent channels would not be expected to improve detection to this degree. It is important to highlight that the NIR-GMM-GAN-UnetD model showed superior performance compared to the previous study [11], which employed the fusion of SWIR and VNIR modalities for FM detection, achieving a remarkable improvement of 8.1% in terms of the JI, even when only the NIR spectral data were used.

### 3.4. Image-Level Detection Performance

Table 5 presents an overview of the false positive rate (FPR) performance. The FPR was utilized to measure the extent to which clean fillets were incorrectly predicted as contaminated. Achieving an FPR of 0, as observed in every NIR and 3PC model, indicates a high level of accuracy in FM detection at the image level. It is worth noting that while the NIR-GAN-EncD model resulted in the addition of false positive pixels, it still achieved an FPR score of 0 since these misclassified pixels were only present in the contaminated samples. With the exception of SWIR, all the other models also achieved an IFPR (incorrect fillet positive rate) score of 0, thereby supporting the effectiveness of the threshold selection method at reducing false positives. Additionally, the results suggest that the proposed method can be applied to various fillet images with different contamination levels, indicating its potential for generalizability.

### 3.5. Limitations and Future Work

This paper acknowledges some limitations and suggests potential directions for future work. One limitation is the incomplete implementation of optimized hyperparameter tuning for the algorithm, which may affect its performance and accuracy across different scenarios. Therefore, future research should focus on a more systematic and rigorous approach to hyperparameter tuning to ensure optimal results. Additionally, the proposed framework was primarily evaluated on chicken breast fillets with FMs on their surface. This means that its performance on other types of poultry products or in scenarios where the FMs are located on the bottom surface of the products remains unclear. It would be necessary to conduct further investigations and evaluations specifically targeting these scenarios. Furthermore, the analysis excluded transparent materials such as glass, which could impact the generalizability of the findings. Additionally, the dataset used in this study was imbalanced, particularly due to the limited number of spectral responses for fat in the chicken breast samples. This imbalance may affect the accuracy and generalizability of the results. To address this limitation, a larger and more balanced dataset would be beneficial, enabling more robust and comprehensive conclusions. For necessary future work, it would be beneficial to focus on further optimizing the algorithm and exploring parallel computing techniques. By improving the algorithmic performance and leveraging the parallel processing capabilities, the real-time detection of FMs can be achieved, expanding the practicality and applicability of the proposed method.

## 4. Conclusions

This study demonstrated a novel semisupervised deep learning approach for foreign material detection in hyperspectral images of poultry meat. The method achieved high accuracy at detecting diverse foreign material types within the hyperspectral images. The proposed GAN effectively addressed the challenge of limited FM data. The detection model optimized only for the NIR wavelengths attained a 96.5% Jaccard Index for blob detection and a 96.8% F1 score at the pixel level, highlighting its potential for practical applications in foreign material detection and other food product quality-assessment tasks. This study set a new paradigm for food contaminant detection by using semisupervised deep learning. The combined spectral clustering, synthetic spectrum generation, and U-Net approach maximized the anomaly detection accuracy while minimizing the annotation requirements. The lightweight GAN discriminator for inferencing unlocks the potential for real-time hyperspectral inspections. In the future, this methodology can be extended to a broader array of food products and contaminant types by assembling larger multiclass datasets. Further optimizations to enhance the computational efficiency would facilitate progress toward seamless deployment in in-line poultry processes. The findings contribute significantly to the field of hyperspectral analysis and its applications in safeguarding the food supply chain.

## Figures and Tables

**Table 1:** Comparison of FID scores of synthetically generated spectra from various GAN models based on two discriminator types (U-Net: UnetD vs. encoder-only: EncD) and three wavelength ranges (SWIR, NIR, and 3PC) compared to the ground-truth SWIR, NIR, and 3PC data. The evaluation of the GAN models’ generative power by using FID scores was based on the spectral clusters of all normal breast pixels (no clustering), only muscle tissue pixels (muscle channel), and only fatty tissue pixels (fat channel).

Clustering	GAN-UnetD				GAN-EncD			
	SWIR	NIR	3PC	Average	SWIR	NIR	3PC	Average
No clustering	2.57	2.14	1.30	2.00	3.73	2.32	1.54	2.53
Muscle channel	2.31	1.32	1.11	1.58	2.49	1.40	1.28	1.72
Fat channel	3.73	3.67	1.70	3.03	3.10	2.76	1.63	2.50
**Average**	2.87	2.38	1.37		3.11	2.16	1.48	

**Table 2:** Summary of FM-detection performance at the pixel level for GAN models using two different discriminators, (**a**) UnetD and (**b**) EncD, over three different spectral ranges (SWIR, NIR, and 3PC) and with and without GMM-based spectral clustering.

**(a)**	**GMM-GAN-EncD**				**No Clustering-GAN-UnetD**				**Overall**
	**SWIR**	**NIR**	**3PC**	**Average**	**SWIR**	**NIR**	**3PC**	**Average**	**Average**
Precision	0.990	1	1	0.997	0.983	1	1	0.994	0.996
Recall	0.939	0.939	0.678	0.852	0.792	0.808	0.678	0.759	0.806
F1 score	0.964	0.968	0.808	0.913	0.877	0.894	0.808	0.860	0.887
BACC	0.970	0.969	0.839	0.926	0.896	0.904	0.839	0.880	0.903
**(b)**	**GMM-GAN-EncD**				**No Clustering-GAN-EncD**				**Overall**
	**SWIR**	**NIR**	**3PC**	**Average**	**SWIR**	**NIR**	**3PC**	**Average**	**Average**
Precision	0.992	1	1	0.997	0.989	0.782	1	0.924	0.961
Recall	0.852	0.823	0.620	0.765	0.739	0.637	0.573	0.650	0.707
F1 score	0.917	0.903	0.765	0.862	0.846	0.702	0.729	0.759	0.810
BACC	0.926	0.911	0.810	0.882	0.870	0.815	0.787	0.824	0.853

**Table 3:** Summary of FM blob detection.

Discriminator Model	Performance Metric	Material Type	GMM-GAN			No Clustering GAN		
			SWIR	NIR	3PC	SWIR	NIR	3PC
UnetD	JI	All	0.839	0.965	0.628	0.846	0.861	0.538
	ODACC	All	0.938	0.965	0.628	0.867	0.861	0.538
	AIoU	Wood	0.899	0.929	0.596	0.865	0.887	0.553
	AIoU	Polymer	0.860	1.000	0.801	0.713	0.750	0.751
	AIoU	Metal	0.921	0.934	0.537	0.931	0.869	0.512
EncD	JI	All	0.772	0.922	0.551	0.850	0.756	0.598
	ODACC	All	0.905	0.922	0.551	0.835	0.822	0.598
	AIoU	Wood	0.918	0.875	0.583	0.828	0.751	0.549
	AIoU	Polymer	0.879	0.915	0.734	0.669	0.924	0.869
	AIoU	Metal	0.907	0.961	0.489	0.867	0.570	0.519

**Table 4:** Detection performance of individual cluster channels and combined detection for blob detection on GAN-UnetD.

Performance Metric	Material Type	SWIR			NIR		
		GMM (FatCh)	GMM (MusCh)	GMM (Proposed)	GMM (FatCh)	GMM (MusCh)	GMM (Proposed)
JI	All	0.680	0.786	0.839	0.588	0.876	0.965
ODACC	All	0.771	0.851	0.938	0.588	0.876	0.965
AIoU	Wood	0.897	0.886	0.899	0.868	0.901	0.929
AIoU	Polymer	0.735	0.716	0.860	0.913	0.800	1.000
AIoU	Metal	0.511	0.842	0.921	0.524	0.881	0.934

**Table 5:** Summary of image-level FM-detection performance measured by using image-level false positive rate (IFPR) for various GAN models including GMM-GAN-UnetD, GAN-UnetD (no clustering), GMM-GAN-EncD, and GAN-EncD (no clustering) across three different spectral ranges (SWIR, NIR, and 3PC). An IFPR of 0 indicates that no false positive errors were observed during the test phase with all normal breast fillet images in the test set.

GMM			No Clustering			GMM			No Clustering		
GAN-UnetD			GAN-UnetD			GAN-EncD			GAN-EncD		
SWIR	NIR	3PC	SWIR	NIR	3PC	SWIR	NIR	3PC	SWIR	NIR	3PC
0.083	0	0	0.083	0	0	0.167	0	0	0.167	0	0

## Data Availability

The data presented in this study are available upon request from the corresponding author.
